# Size limits of magnetic-domain engineering in continuous in-plane exchange-bias prototype films

**DOI:** 10.3762/bjnano.9.276

**Published:** 2018-12-03

**Authors:** Alexander Gaul, Daniel Emmrich, Timo Ueltzhöffer, Henning Huckfeldt, Hatice Doğanay, Johanna Hackl, Muhammad Imtiaz Khan, Daniel M Gottlob, Gregor Hartmann, André Beyer, Dennis Holzinger, Slavomír Nemšák, Claus M Schneider, Armin Gölzhäuser, Günter Reiss, Arno Ehresmann

**Affiliations:** 1 Institute of Physics and Center for Interdisciplinary Nanostructure Science and Technology (CINSaT), University of Kassel, 34132 Kassel, Germany; 2 Faculty of Physics, Physics of supramolecular Systems and Surfaces, University of Bielefeld, 33501 Bielefeld, Germany; 3 PT-DESY, Deutsches Elektronen-Synchrotron DESY, 22607 Hamburg, Germany; 4 Peter Grünberg Institute “Electronic Properties”, Forschungszentrum Jülich, 52425 Jülich, Germany; 5 Advanced Light Source, Lawrence Berkeley National Laboratory, Berkeley, CA, USA; 6 Faculty of Physics, Thin Films and Physics of Nanostructures, University of Bielefeld, 33501 Bielefeld, Germany

**Keywords:** exchange bias, helium ion microscopy, ion bombardment induced magnetic patterning, magnetic domains, magnetic nanostructures

## Abstract

**Background:** The application of superparamagnetic particles as biomolecular transporters in microfluidic systems for lab-on-a-chip applications crucially depends on the ability to control their motion. One approach for magnetic-particle motion control is the superposition of static magnetic stray field landscapes (MFLs) with dynamically varying external fields. These MFLs may emerge from magnetic domains engineered both in shape and in their local anisotropies. Motion control of smaller beads does necessarily need smaller magnetic patterns, i.e., MFLs varying on smaller lateral scales. The achievable size limit of engineered magnetic domains depends on the magnetic patterning method and on the magnetic anisotropies of the material system. Smallest patterns are expected to be in the range of the domain wall width of the particular material system. To explore these limits a patterning technology is needed with a spatial resolution significantly smaller than the domain wall width.

**Results:** We demonstrate the application of a helium ion microscope with a beam diameter of 8 nm as a mask-less method for local domain patterning of magnetic thin-film systems. For a prototypical in-plane exchange-bias system the domain wall width has been investigated as a function of the angle between unidirectional anisotropy and domain wall. By shrinking the domain size of periodic domain stripes, we analyzed the influence of domain wall overlap on the domain stability. Finally, by changing the geometry of artificial two-dimensional domains, the influence of domain wall overlap and domain wall geometry on the ultimate domain size in the chosen system was analyzed.

**Conclusion:** The application of a helium ion microscope for magnetic patterning has been shown. It allowed for exploring the fundamental limits of domain engineering in an in-plane exchange-bias thin film as a prototypical system. For two-dimensional domains the limit depends on the domain geometry. The relative orientation between domain wall and anisotropy axes is a crucial parameter and therefore influences the achievable minimum domain size dramatically.

## Introduction

Engineered magnetic domains with deliberately set magnetic properties and designed shapes in thin-film systems have proven to be useful in memory [1–2] and sensor applications [3–5], for stray field design [6–7] and particle transport in lab-on-chip systems [8–11], or in spintronics and magnonics [12–14]. Currently available techniques for domain patterning are either based on focused ion beams (FIB) [15–17], ion implantation [18–21], laser annealing [22–24], thermally assisted scanning probe lithography [25], or a combination of spatially broad laser- or ion-beams and shadow masks [26–30]. Especially in magnonic [14] and sensor applications [4] in-plane magnetic domain patterns play a key role and are one of the objectives of recent research to create tailored domain shapes on the one hand and to minimize the domains to the nanometer regime on the other hand.

The size limit of patterning magnetic domains in continuous in-plane layer systems is expected to be in the range of the domain wall (DW) width, varying with material-specific magnetic parameters, but could not be tested yet. For exchange-bias material systems with in-plane anisotropy, typical DW widths are of the order of several hundreds of nanometers [16] to some micrometers [31]. A patterning method with lateral resolution significantly smaller than the domain wall width and a characterization method with sufficient spatial resolution are required to investigate this size limit and its dependence on the magnetization orientation and intrinsic magnetic properties of a layer system. Except for very few attempts for magnetic patterning by Ga ions in a FIB (suffering from destruction of the thin films due to high sputter yields) [32–33], available patterning methods do not achieve the necessary resolution. Currently, the smallest engineered domains in films with in-plane anisotropy are 300 nm wide stripes produced by thermally assisted scanning probe lithography [25] or 250 nm wide dots fabricated by direct interferometric laser annealing [34]. Local annealing, however, results in three-dimensional temperature gradients within the magnetic film causing thermal diffusion and material intermixing over several hundreds of nanometers [25]. Local magnetic property modifications in thin films by narrow beams of light ions, in contrast, do not suffer from this drawback due to more localized energy deposition [35]. Currently patterning by kiloelectronvolt light ion bombardment is performed using shadow masks where the lateral resolution is limited by relatively thick polymer masks in combination with non-optimum edge steepness [36–37]. In addition, electrostatic charging of the mask [27] can lead to further beam broadening resulting in areas of gradually changing ion doses between bombarded and non-bombarded regions.

Thus, at present there is no method available where the lateral resolution is considerably higher as the expected minimum pattern sizes. Here we suggest mask-less patterning by the highly focused beam of a helium ion microscope (HIM), to lower the limits of ion beam induced magnetic pattering in continuous layer systems [38]. The method is demonstrated for engineered domains in one of the most popular and well-examined exchange-bias (EB) layer systems [35,39–41], Ir_17_Mn_83_ (30 nm)/Co_70_Fe_30_ (10 nm), as a prototype with unidirectional in-plane anisotropy 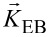, but it can be easily extended to other magnetic material systems.

More specifically, the size limit of thermally stable engineered magnetic domains has been studied for prototypical domain geometries, and for varying magnetization directions with respect to the DWs. The prototypical EB system, with respect to saturation magnetization, magneto crystalline anisotropy and theoretically predicted domain wall width, was chosen to be fully accessed by a variety of quantitative characterization methods. The analysis of the patterns has been achieved by complementary experimental methods, characterizing the magnetization profile by X-ray photo emission electron microscopy (X-PEEM) and investigating the magnetic charge state of the DWs by magnetic force microscopy (MFM). The experiments have been corroborated by micromagnetic simulations.

## Results and Discussion

The ion bombardment induced magnetic pattering of artificial domains in exchange-bias multilayers is fundamentally based on two energy-transfer mechanisms from the ions to the material system. The predominant effect, the electronic energy transfer (hyperthermal heating), causes a reorientation of the local unidirectional anisotropy. The second and considerably weaker effect is the nuclear energy transfer causing defects in the atomic lattice structure [35]. These defects do not change the orientation of the local unidirectional anisotropy, but rather influence the local magnetic properties, as already explained in [35,40].

The area modified by an ion beam is defined by the beam diameter and the ion straggling in the sample. The corresponding lateral range of this effect has been estimated by SRIM [42] simulations to be less than 30 nm at the ferromagnet (F)/antiferromagnet (AF) interface of the investigated layer system (see Appendix, Figure 5) and maximum 90 nm in the deep bulk of the AF. Therefore, an ion beam of 8 nm diameter achieves a patterning width of less than 40 nm at the EB interface well below the expected material specific size limit for stable domains.

This size limit is defined by the DW width between in-plane engineered EB domains and depends on the relative orientations of the unidirectional anisotropies in the adjacent domains and the DW normal vector. Whereas its dependence on the magnetization orientations in adjacent domains was shown recently [43], the dependence on the angle between magnetization and DW normal vector for fixed domain magnetizations in equally shaped domains was not analyzed systematically. The latter is expected to evoke a geometry dependence of the minimum size for engineered domains as the domain geometry will define the relative orientations of the magnetizations in adjacent domains with respect to the DW. In a preparatory experiment DW widths have been investigated as a function of the angle 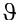 between fixed antiparallel unidirectional anisotropies of adjacent domains and the DW normal vector 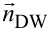 (see Appendix, Figure 6).

**Figure 1:**
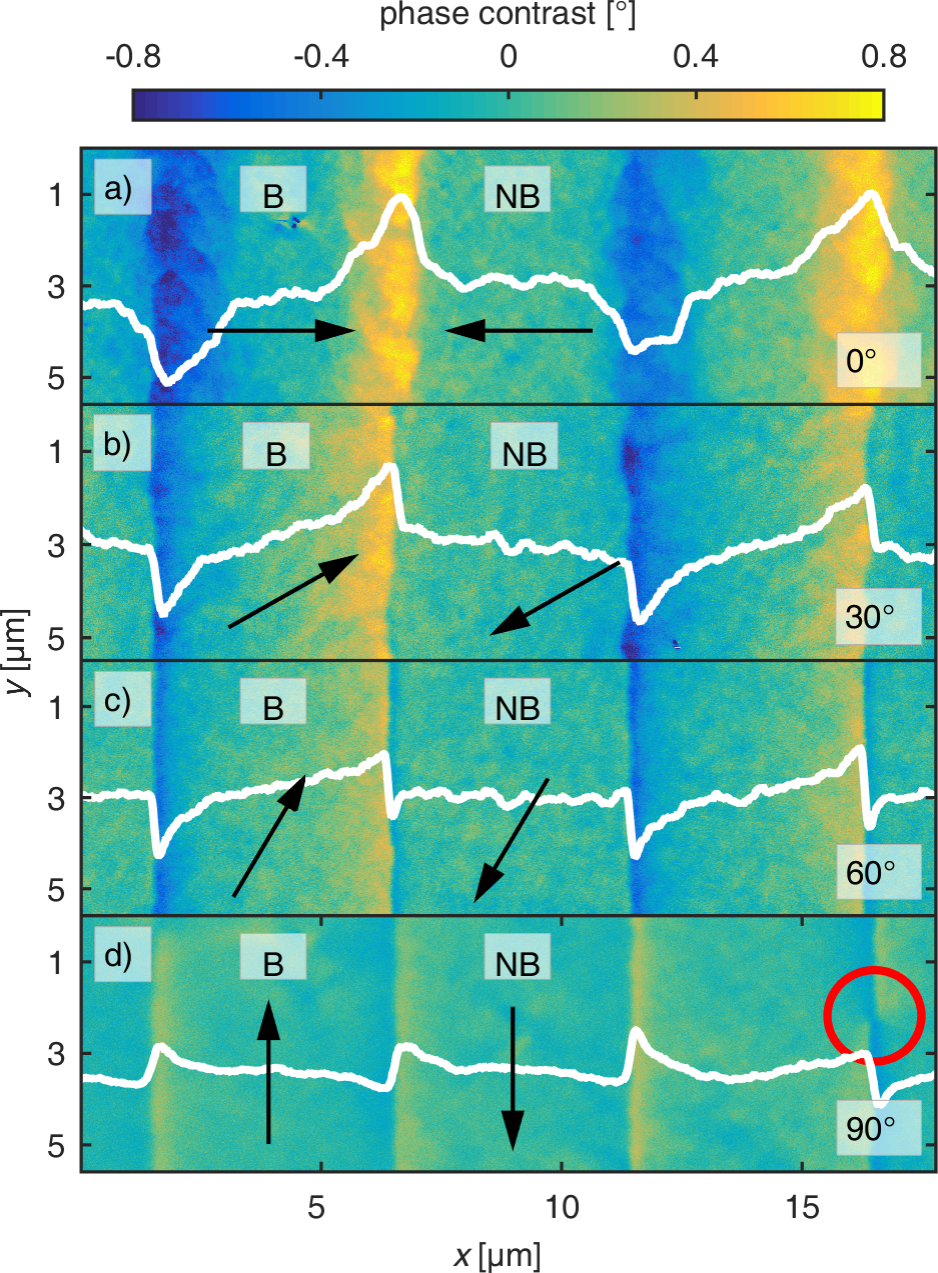
Phase contrast MFM images of engineered parallel-stripe domains. Magnetic domains with antiparallel magnetization orientation have been observed at an MFM tip height of 80 nm as a function of 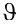, the angle between unidirectional anisotropy and DW normal vector, indicated in the bottom right corner of the images. The white lines are cross sections of the signal along a stripe at a *y*-position of 3 μm averaged over 100 nm of width. Arrows mark the local directions of the unidirectional anisotropies of the bombarded (B) and non-bombarded (NB) stripes. The red circle is highlighting the position of a Bloch point.

For these experiments, 5 μm wide periodic parallel domain stripes with antiparallel unidirectional anisotropies have been fabricated by a slightly defocused 15 keV He-ion beam of 8 nm diameter (Figure 1). For the different stripe patterns, the angle 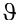 has been varied in increments of 30°. Experimentally, this has been realized by changing the stripe orientation with respect to the initial EB field direction. For these domain geometries, the DW charge state is expected to change from monopolar for head-to-head (hh) and tail-to-tail (tt) domain configurations (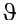 = 0°) to bipolar for the side-by-side (ss) configuration (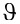 = 90°). The DW charge state in the *xy*-plane, parallel to the F layer, was characterized by MFM (Figure 1). For 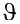 = 0°, the hh and tt domain configuration leads to a maximization of the monopolar charge density within the DW. To reduce the stray-field energy, the DW core spreads into the adjacent domains. Substructures visible in Figure 1a close to the DW center originate from the high charge density in the domain wall center, causing a widening of the latter [44].

The DW spreads wider into the bombarded areas than into the non-bombarded areas, resulting in asymmetric DWs. This is caused by the reduced effective magnetic anisotropy within the bombarded regions correlated to the nuclear ionic effects [6]. The change of the spatial distribution of the charge contrast, when varying 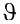 from 0° (Figure 1a) to 90° (Figure 1d), results from the transition of monopolar to bipolar magnetic charge states. This is associated with a decrease of the charge contrast in the center of the DWs. The charge contrast signal within the domains shows a plateau for 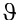 = 0° and 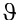 = 90°, while for 0° < 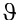 < 90° the signal continuously changes through the whole domain indicating a wider spread of the monopolar DW charges into the domain. This effect is attributed to the misalignment of the uniaxial F anisotropy and the domain wall normal.

The DW widths are crucial for the miniaturization of domains, since the interaction of DWs may destabilize the domain, e.g., by domain wall tail overlap [44]. The data for 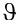 = 0° and 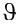 = 90° are in quantitative agreement with prior investigations on lithographically patterned stripe domains [43].

**Figure 2:**
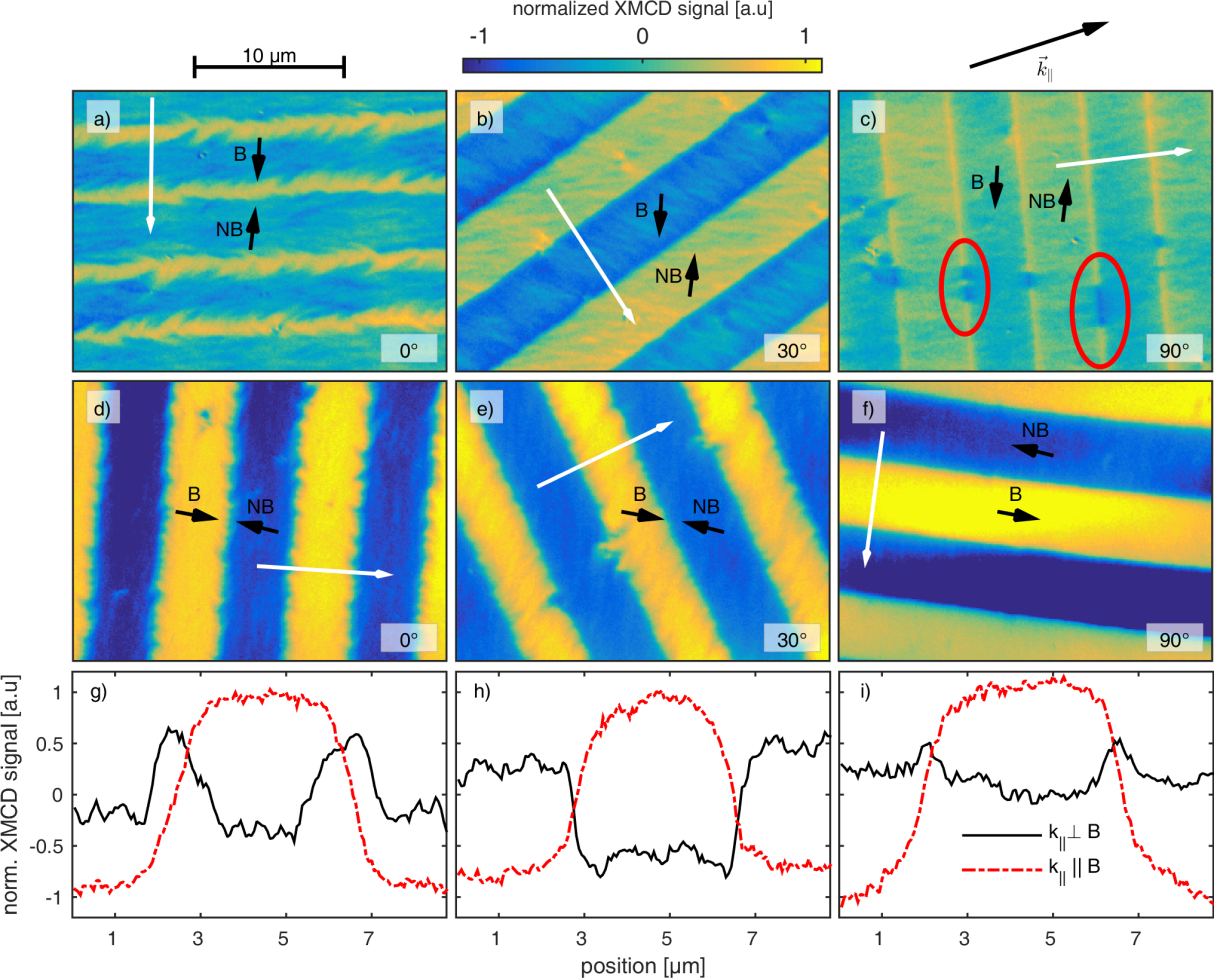
XMCD signal images of engineered parallel domain stripes. Magnetic domains with antiparallel magnetization orientations have been analyzed in dependence on 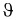. Black arrows mark the directions of the set unidirectional anisotropies in bombarded (B) and non-bombarded (NB) regions. The orientation of sensitivity (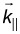) is indicated by the top right black arrow. Red ellipses highlight sign inversions in the DW signal, with the corresponding Bloch points at the margins. White arrows denote cross sections shown in panels (g–i) (position increasing along arrow direction). Black solid lines in panels (g–i) represent measurements with sensitivity perpendicular to 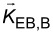 (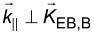) (a–c) and red dash dotted lines those with 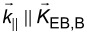 (d–f). Note that the XMCD signal corresponds to cos α where α is the angle between 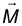 and 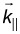.

To complement the MFM data, X-PEEM measurements have been performed to analyze the local magnetization states by X-ray magnetic circular dichroism (XMCD) measurements. Two different sensitivity directions have been chosen: Figure 2a–c shows the results when the projection 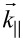 of the impinging X-ray wave vector on the substrate surface is almost perpendicular to the unidirectional anisotropy of the bombarded stripe regions 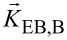 (
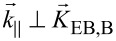
). These data are sensitive to the magnetization orientation within the DW cores. Figure 2d–f depict the results for 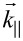 almost parallel to 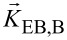 (
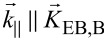
). For hh and tt domain configuration, i.e., 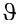 = 0°, the zig-zag-shaped magnetization distribution causing large DW widths is obvious (Figure 2a,g). The angle δ between bombarded and non-bombarded regions was determined to be δ = 184° ± 2° from a mathematical fit to the measured data. This slight misalignment of the engineered unidirectional anisotropy axes causes unwinding DWs [44] over the whole patterned area (yellow DW contrast in Figure 2a and wide maxima in the black line in Figure 2g).

The DWs for the ss domain configuration (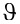 = 90°, Figure 2c,i) appear as narrow stripes with lower maximum peak values, indicating less magnetic moments oriented parallel to the DW normal. Detailing the DW contrast in Figure 2c (red ellipses) a signal sign inversion of the DW indicates a change of the rotation sense of the DW (Bloch point) [44]. Bloch points are also visible in the MFM data for bipolar charged DWs (e.g., Figure 1d, red circle). The stripe pattern with 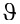 = 30° (Figure 2b,h) shows almost no DW signal in the border regions of the pattern. However, it becomes clear from the intersection profiles along the white arrows (Figure 2h), that the signal difference between bombarded and non-bombarded domains is well pronounced (black line, Figure 2h) and much higher than for 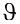 = 0° and 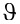 = 90°, indicating that the magnetization direction within these regions is not parallel aligned with respect to the engineered unidirectional anisotropy direction. This corroborates the finding of wide DW tails into the domains, caused by the misalignment between DW and unidirectional anisotropies.

The results of Figure 2a–c are further supported by the results displayed in Figure 2d–f and the profiles along the white arrows (red lines in Figure 2g–i), recorded for 
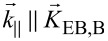
. Also, here the signal contrast is not evenly distributed within the domains and it is associated with the tails of Néel-type DWs. Again, the images for the hh and tt domain configurations support the presence of zig-zag DWs vanishing for decreasing monopolar charge densities, i.e., 0° < 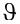 ≤ 90°.

Based on these results, we have successively decreased the widths of parallel-stripe domains for the hh and tt magnetization configuration where DWs are carrying maximum magnetic net charges. This configuration was chosen since maximum net charges are a limiting factor for the minimization of artificial domains. For this purpose, sets of identical parallel-stripe domains, with engineered widths *b* of 5 μm, 2 μm, 1 μm, 500 nm, 200 nm and 100 nm were written by HIM in an external magnetic field, applied antiparallel to the initial EB field. For *b* ≥ 500 nm, the stripe repetition number was chosen to be *N* = 5, whereas for *b* < 500 nm, *N* = 10.

**Figure 3:**
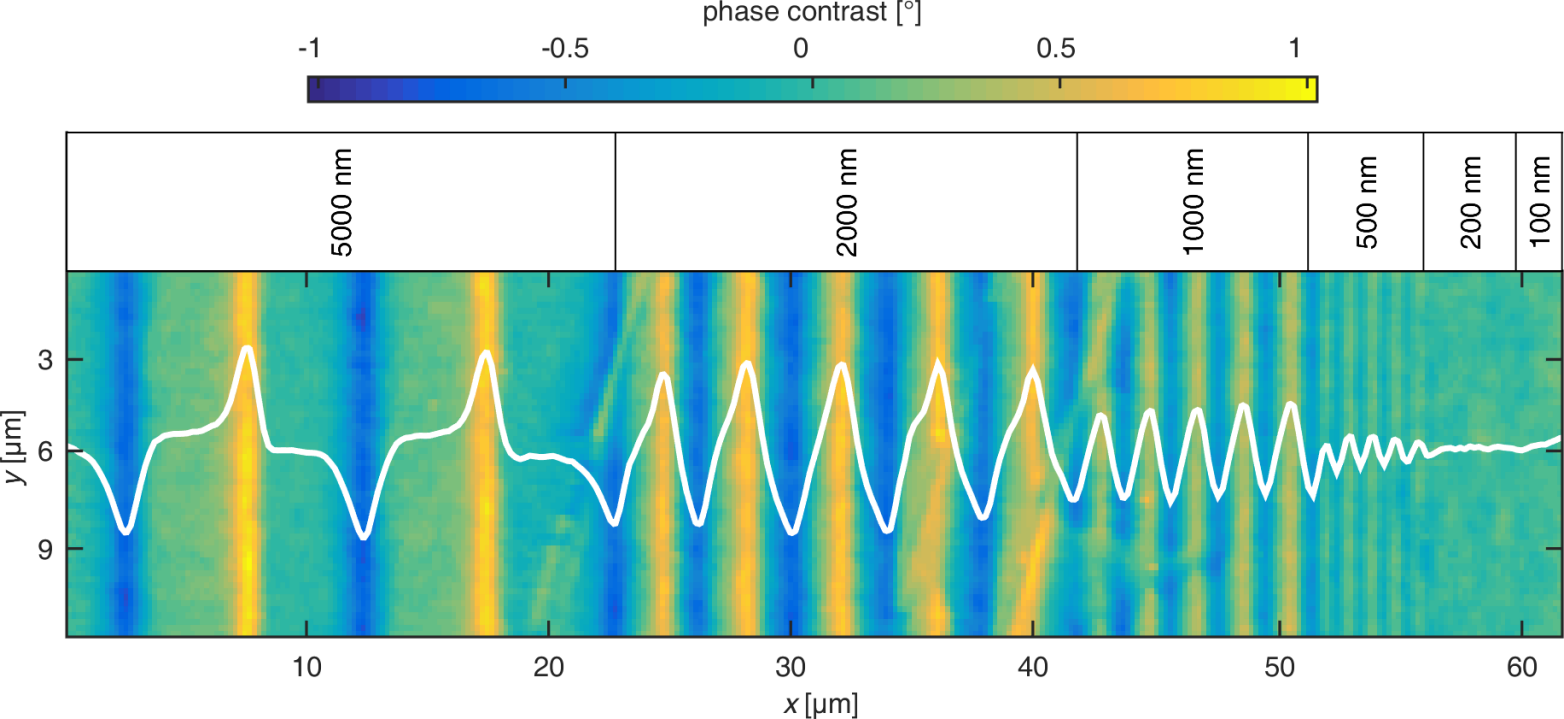
Phase contrast MFM signal of domains with stepwise decreased nominal widths and hh and tt magnetization orientations. The measurement height was set to 100 nm. The white line indicates a cross section of the phase contrast signal along a stripe at a *y*-position of 6 μm averaged over 4 μm width. The black boxes above the measurement data highlight the position of the different areas containing set stripe patterns with widths of 5 μm, 2 μm, 1 μm, 500 nm, 200 nm and 100 nm.

The magnetic charge contrast of this pattern obtained by MFM is shown in Figure 3. For stripe domains with *b* = 5 μm, the DW signals can be clearly distinguished from those of the domain center above which the measured phase contrast is almost zero. This finding reproduces the results from Figure 1a. When decreasing *b*, these plateau-like regions vanish, i.e., DWs and domains can no longer be distinguished as there is a continuous transition between neighboring DWs of inverted charge. For our prototypical system, this is the case for *b* ≤ 2 μm (Figure 3). Since the theoretically predicted DW tails, calculated from [31] with the modification of the saturation magnetization [35] and the uniaxial magnetic anisotropy constant [40] are *D*_tail,B_ = 1.32 μm for the bombarded and *D*_tail,NB_ = 1.04 μm for the non-bombarded regions (see Appendix for details), it is evident that for *b* ≤ 2 μm there is a significant crosstalk between neighboring DWs. However, the smallest distinguishable periodic magnetic patterns are observable in Figure 3 for *b* = 500 nm. In earlier experiments using masks, a critical domain width of 700 nm has been found for non-periodic domain patterns with hh and tt in a Fe_50_Mn_50_ (10 nm)/Ni_81_Fe_19_ (5 nm) layer system [29]. Although the magneto-crystalline anisotropy of Ni_81_Fe_19_ is smaller than that of Co_70_Fe_30_, it is the small saturation magnetization *M*_s_ together with a thinner F layer that potentially allows for the observation of smaller DW tails in this material system (*D*_tail,B_ = 972 nm in bombarded regions; *D*_tail,NB_ = 874 nm in non-bombarded regions, see Appendix) and therefore smaller thermally stable domains. The limitations correlated to the patterning process when using shadow masks, however, cause spatial broadening of the ion dose gradient. Since the concerned regions correspond to the DW regions, there is a strong impact on the actual DW fine structure due to both geometrical and magnetic deviations from the set structure leading to a larger minimum domain size.

**Figure 4:**
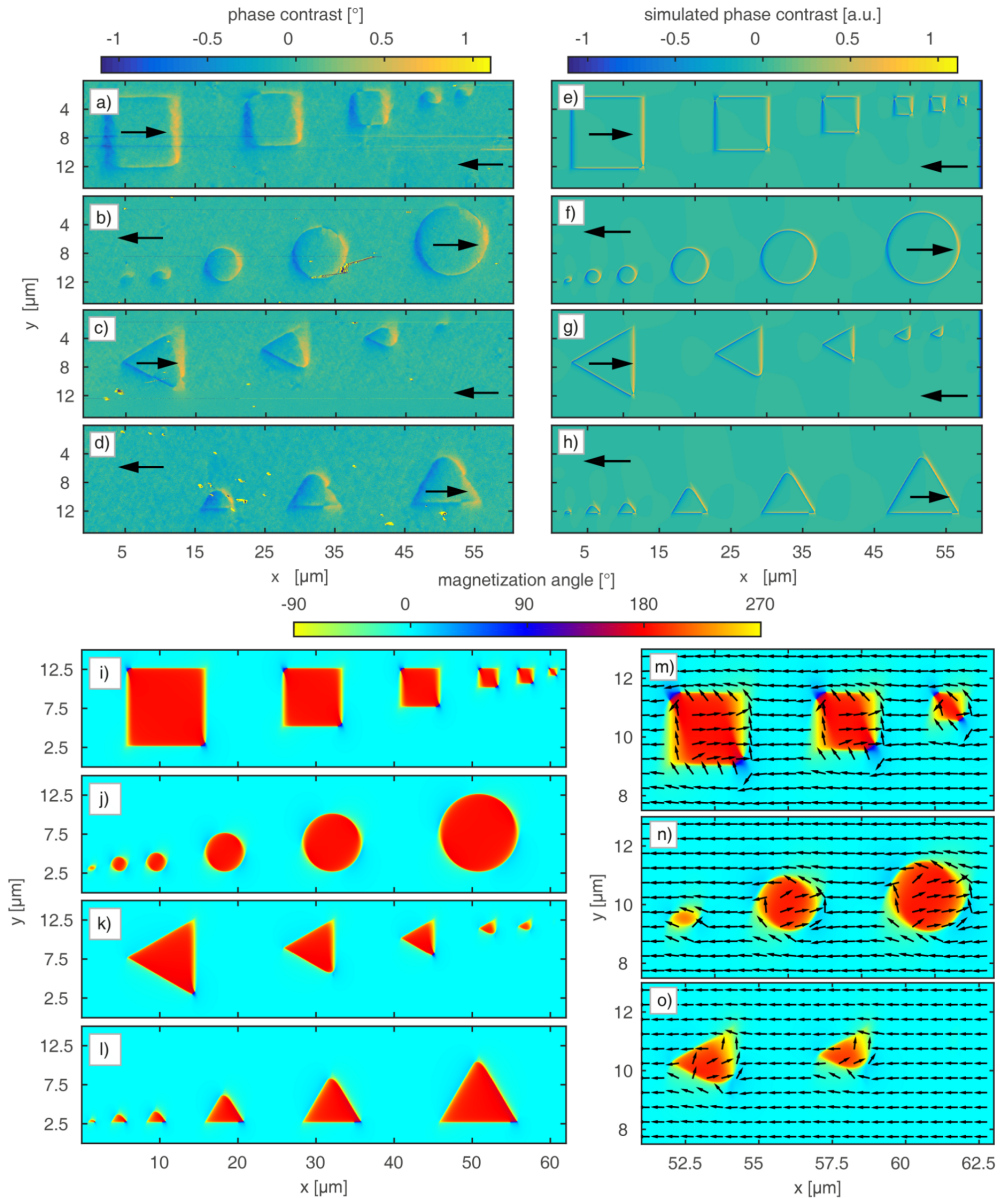
Two-dimensional engineered magnetic domain patterns. (a–d) Phase contrast MFM images in 80 nm height of engineered magnetic patterns with stepwise decreased edge length *d* = 10 μm (largest domain: either left-most or right-most), 7.5 μm, 5 μm, 2.5 μm, 2 μm and 1 μm. (e–h) Calculated phase contrast from the simulations in panels (i–l). Black arrows indicate the local direction of the unidirectional anisotropy in panels (a–h). (i–o) Simulated magnetization distributions from micromagnetic simulations in OOMMF. Colors depict the local *xy*-magnetization angle where 0° represents the initial EB direction pointing to the left, positive angles imply counterclockwise rotation. (m–o) Magnified view on the smallest domain structures from panels (i–l). Arrows indicate the direction (orientation) and relative value (length) of the local magnetic moment.

To determine the ultimate size limit of the prototypical EB-system, the results of the previous studies were merged by a series of experiments with two-dimensional domains of three fundamental shapes. These shapes, namely squares, circles, and equilateral triangles, with deliberately set edge lengths or diameters *d* of 10 μm, 7.5 μm, 5 μm, 2.5 μm, 2 μm, 1 μm and 500 nm, comprise the previously investigated different angles 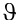 and different DW–DW distances. The domain patterns were analyzed by MFM and simulated using the object-oriented micromagnetic framework (OOMMF) [45] (Figure 4).

While the square-shaped domains (Figure 4a) are surrounded by DWs with 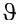 = 0° and 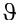 = 90°, DWs of the circular domains (Figure 4b) are characterized by continuous transitions of their charge state from 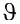 = 0° (left and right border) to 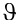 = 90° (upper and lower border) and back. For the equilateral triangles two exemplary patterns have been realized: one with the symmetry axis parallel (Figure 4c,g) and one with the symmetry axis perpendicular (Figure 4d,h) to the unidirectional anisotropy axis of the layer system, resulting in DWs with 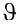 = 0° and 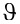 = 60° (Figure 4c,g) and 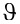 = 30° and 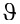 = 90° (Figure 4d,h), respectively. Figure 4 shows that the smallest stable domains of the prototypical system are obtained for square and circular domains of *d* = 2 μm and *d* = 5 μm for the triangular domains, where the actual domain shape shows significant distortion at the vertices independent of the domain size. The increased minimum domain size of the triangular structures is caused by the overlap of the longer DW tails of 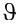 = 30° and 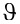 = 60° DWs in comparison to ss or hh/tt DWs as described before.

For charged DWs it is a priori not possible to correlate the MFM signal to the magnetization configuration, as there is no one-to-one correspondence of these two quantities [43]. Therefore, we performed micromagnetic simulations in OOMMF for the domain shapes of Figure 4a–d. The resulting spatial magnetization distribution of the F layer (Figure 4i–o) was used as an input parameter to calculate MFM images within the limit of negligible interaction between tip and sample and for a uniformly magnetized MFM tip. The results of the simulations are shown in Figure 4e–h. The MFM signal generated by DWs of different charge states is qualitatively reproduced. hh and tt DWs carrying a monopolar charge appear with comparably strong signals either with positive or negative sign (Figure 4a,e) while dipolar charged ss DWs show bipolar charge contrast in agreement to the experiment (Figure 1a,d). The substructures present for DWs with hh and tt configurations caused by local fluctuations of the demagnetization field are not reproduced by the simulations. This deviation is attributed to the polycrystallinity of the layer system, which is not included in the simulations. As a result, the fluctuations of material parameters combined with local angular fluctuations of the anisotropy axes typically lead to the formation of ripple structures [44], i.e., periodic fluctuations of the local magnetization orientation. Therefore, magnetic charges are also generated within the domains, which lead to a reduction of the overall magnetic charge density minimizing the stray-field energy. These ripple structures are responsible for the significant spatial broadening of the experimentally observed charge profile when compared to the simulations. The shape of the smallest stable quadratic domain (*d* = 2 μm) also appears distorted in the MFM data and the different DW types appear blurred. In the simulations, such structures are clearly visible and blurring of DWs appears at *d* = 1 μm. Again, the reason for the discrepancy between measurement and simulation can be attributed to local fluctuations of material parameters resulting from the polycrystallinity of the layer system and the sample–tip distance. Also, the magnetization profile of the MFM tip was neglected in the simulations.

Since simulations and measurements are in very good qualitative agreement concerning DW types and distortion, the simulated magnetization distributions (Figure 4i–o) are used to further interpret the experimental data. From the simulated domain configuration (Figure 4i) it is apparent that the simulated slight misalignment of the adjacent magnetizations (δ = 183.9°) promotes an almost uniform magnetization orientation in the DWs, visible as a prevailing yellow DW contrast. This is in accordance with the XMCD data in Figure 2, where the misalignment of the adjacent unidirectional anisotropy axes promoted unwinding DWs. The corresponding charges are compensated by opposite magnetization areas in the top-left and bottom-right corners (blue areas). From Figure 4m, it is evident that the interplay of magnetic charge based stray fields and local anisotropy causes the magnetization rotation to reach deep into the magnetic domains. The domain center is not completely oriented along 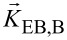; instead the domain shows a curled magnetization state. Magnetic charges of inverted polarity are, therefore, close together and cannot be detected by the MFM tip, averaging over a certain lateral range defined by the tip radius and the distance to the sample surface.

The DW profile obtained for circular domains in the simulations also qualitatively reproduces the measurement. The simulations underestimate the smallest stable domain size because of the discussed reasons. In contrast to the triangular domains, the minimum size of circular domains is smaller: as the DW charge state continuously changes along the domain boundary corresponding to the radial rotation of the DW normal vector, the resulting demagnetization field changes as well. Thus, the torque that results from the interaction between the demagnetization field and the magnetization distribution of the domain decreases radially and leads to a more uniform orientation of the magnetization field (Figure 4j,n) within the domains.

For the triangular domains (Figure 4c,d), the observed charge contrast is again supported by the simulations (Figure 4g,h) and the MFM data of Figure 1. The experimentally observed distortions of the vertices are also visible in the simulations (Figure 4o). Moreover, the observed DW structure is reproduced qualitatively in the simulations except for their widths appearing wider in the experiments. This difference is attributed to the signal averaging due to the MFM tip size, to the polycrystalline fine structure of the layer system causing local anisotropy fluctuations [43] and to the fact that the unidirectional anisotropy of the EB has been mimicked by a local magnetic field. Since the DW charge states remain unaffected along the DW, a strong interaction between the corresponding demagnetization fields is present in the regions close to the domain vertices. As a result, the influence of the demagnetization field leads to the formation of a local, almost flux-closure-like pattern of the magnetic moment distribution. This can be also seen in the simulations particularly for the smaller domains, since there the contribution of the magnetocrystalline anisotropy energy is comparably stronger [29]. For triangular domains with symmetry axes parallel to the unidirectional anisotropy axis, the simulations predict a smallest stable domain size of *d* = 2 μm, with already strong alteration of the set domain shape. This value is, for the abovementioned reasons, smaller than experimentally found (*d* = 5 μm).

## Conclusion

By employing the helium ion beam of a scanning helium ion microscope defocused to 8 nm for mask-less ion bombardment induced magnetic patterning a prototypical in-plane exchange-biased layer system has been modified locally with a resulting patterned spot of less than 40 nm diameter at the AF/F interface. The narrow beam diameter enabled lateral magnetic modifications of the continuous layer system well below currently available light ion patterning techniques and well below expected stable domain sizes. It was shown that the domain wall width is strongly connected to the angle between unidirectional anisotropy and domain wall normal. Additionally, the influence of the domain wall overlap on the domain stability was quantified. For magnetic-domain engineering, this method enables strategies to fabricate domains of minimum size. It was shown that the minimum domain size for magnetic stripes with head-to-head magnetization configuration in the chosen prototypical system is at least 500 nm. For two-dimensional domains, the minimum stable domain size depends on the domain shape and the interplay of both domain and domain wall charges, corresponding stray fields and local anisotropies. For the presently investigated thin film system with in-plane anisotropy, key enablers to achieve minimum domain sizes are rounded vertices to support continuous charge transitions, the avoidance of monopolar charges and of DWs with 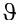 ≠ 0° or 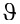 ≠ 90°. The smallest domain size was found for square and circular structures to be 2 µm.

This method allows for the magnetic patterning via kiloelectronvolt light ion beams of a variety of material systems in order to test for fundamental properties governing minimum achievable domain sizes.

## Experimental

### Sample preparation

The prototypical in-plane EB layer system Ir_17_Mn_83_ (30 nm)/Co_70_Fe_30_ (10 nm) was grown by RF sputtering at a power of 160 W and an argon gas flux of 155 sccm on a naturally oxidized 5 nm × 5 nm Si(100) wafer, with Cu (5 nm) buffer and Ta (10 nm) capping. EB at the interface between the antiferromagnetic (AF) and ferromagnetic (F) layer has been initialized by heating at 573 K for 90 min and subsequent cooling at a rate of 1 K·min^−1^ for 300 min to room temperature in an external magnetic field of 80 kA·m^−1^.

### HIM patterning

A commercial HIM (Zeiss Orion Plus) has been modified with a sample holder allowing for the application of an in-plane magnetic field of 95 kA·m^−1^ during ion bombardment. The samples were aligned with their initial EB-field direction pointing antiparallel to the external magnetic field of the holder. 15 keV helium ion bombardment was performed on an area of 500 μm × 500 μm consisting of 2^16^ × 2^16^ separate points. To do so, the ion beam was defocused, leading to a probe diameter of 8 nm. The resolution was determined by the knife-edge method from the image sharpness [46]. The ion dose was chosen to be 1 × 10^15^ ions·cm^−2^ to induce a maximum change of *H*_EB_ [47]. A Raith Elphy multibeam pattern generator was used to write the designed domain shapes and patterns within the continuous thin film.

### MFM characterization

MFM measurements were performed by a Nanosurf Flex-AFM with C3000 controller in tapping/lift mode with a lift height of 80 nm, a peak-to-peak amplitude of 80 nm and a pixel size of 20 nm. These settings were chosen from preliminary experiments as a trade-off between lateral charge-contrast resolution and minimal signal overlap from the sample topography. Additionally, an SIS ULTRAObjective in non-contact/lift mode with a lift height of 100 nm and a pixel size of 200 nm was applied for the MFM measurements. Hard magnetic MFM probes (Nanosensors PPP-MFMR) with a nominal resonance frequency of 70 kHz and a spring constant of 2.8 N·m^−1^ were employed.

### XPEEM characterization

XPEEM measurements [48–49] of the local magnetization distribution 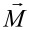 in the F layer of the EB layer system were performed at beamline UE56/1-SGM of the synchrotron radiation facility BESSY II after excitation with right and left circularly polarized X-rays with an energy of 709 eV (Fe L_3_ edge). Prior to measurements, the capping layer was thinned by argon ion sputtering allowing the Fe photoelectrons to escape from the layer system. The XPEEM measurements were carried out in grazing incidence of the incoming synchrotron radiation while the angle between the photon 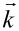-vector surface projection 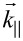 and the initially set EB field direction of the thin film was varied. Measurements of partial electron yield maps were imaged with a 43 μm field of view. One image pixel represents a sample area of 65 nm × 65 nm. Of particular note is that the XMCD signal Δ of the partial electron yield of the two respective helicities (Δ = (*I*_σ+_ − *I*_σ−_)/(*I*_σ+_ + *I*_σ−_)) is proportional to 
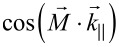
.

### Micromagnetic simulations

Micromagnetic simulations were carried out using the object-oriented micromagnetic framework (OOMMF) for a 10 nm thick Co_70_Fe_30_ film assuming a uniaxial magnetic anisotropy constant of *K*_F,NB_ = 4.5 × 10^4^ J·m^−3^ for the non-bombarded areas [43] and *K*_F,B_ = 0.71*K*_F,NB_ for the bombarded areas [40]. The saturation magnetization in the non-bombarded areas was chosen to be *M*_s,NB_ = 1226 kA·m^−1^ and *M*_s,B_ = 1175 kA·m^−1^ for the ion-bombarded regions [35]. The exchange constant was chosen to be *A* = 3 × 10^−11^ J·m^−1^ [50]. The EB field within the non-bombarded (*H*_EB,NB_ = 12.3 kA·m^−1^) and bombarded areas (*H*_EB,B_ = 10.0 kA·m^−1^) has been mimicked by a local magnetic field the field direction of which corresponds to the direction of the unidirectional anisotropy of the domain. EB fields were determined by Kerr microscopy of the engineered domain pattern. The mesh size of 10 nm × 10 nm was chosen to follow the stray-field exchange length of the film of 6 nm [44,51]. Based on the XMCD data, the angle δ between the initial EB field direction and the EB field within the bombarded regions was chosen to be δ = 183.9°. The simulations were accomplished by conjugate gradient minimization of the local torque 
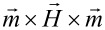
 between the unit magnetization direction 
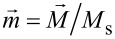
 of one mesh element and the local total magnetic field 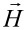 starting from an ideal alignment of the set magnetization direction along the EB field direction. Note that on basis of experimental findings *M*_s_ was chosen differently for bombarded and non-bombarded areas [35]. The stopping condition for the simulations was set for 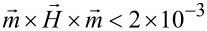 or a maximum step number of 2 × 10^5^ iterations.

## Appendix

**SRIM simulations of the ion energy loss distribution:** To determine the distribution of ions and the spatial distribution of the transferred energy in the sample, simulations using the SRIM software framework have been performed [42]. The layer system has been modeled with its nominal thicknesses and the following material densities: ρ_Si_ = 2.32 g·cm^−3^ [52], 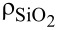 = 2.65 g·cm^−3^ [52], ρ_Cu_ = 8.92 g·cm^−3^ [52], 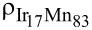 = 8.386 g·cm^−3^ [52], 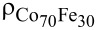 = 8.565 g·cm^−3^ [52], ρ_Ta_ = 16.65 g·cm^−3^ [52]. The densities 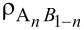 of binary alloys with A*_n_*B_1−_*_n_* stoichiometry were approximated from the density values of pure metals using


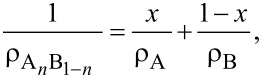


with x being the mole fraction, *x* = *n*/100. The SRIM compound correction was deactivated for all layers except SiO_2_.

**Figure 5:**
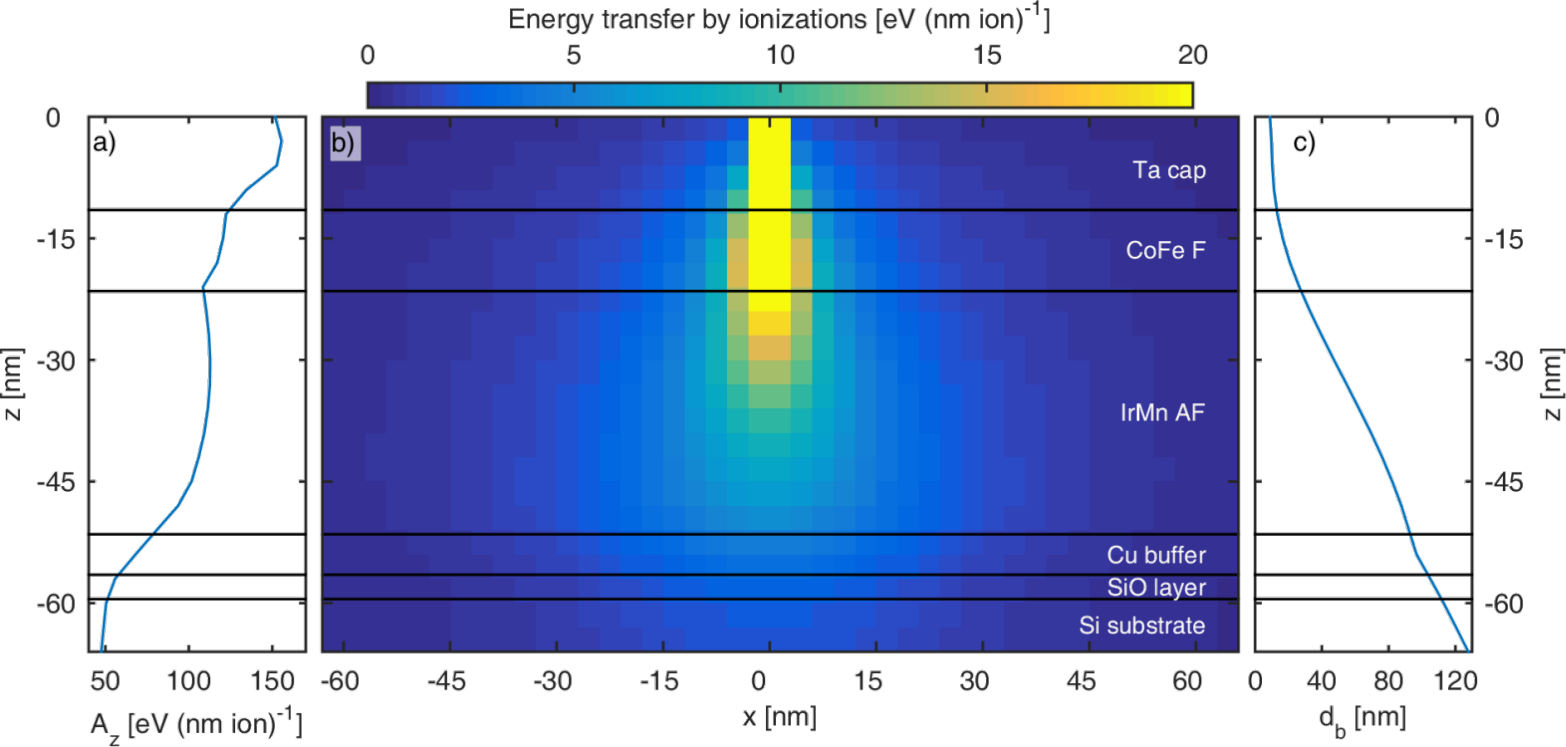
Simulated distribution of the energy transfer by ionization per ion. The positions of layer borders are indicated by black lines. (a) Energy loss *A_z_* as a function of the penetration depth *z*. (b) Spatial energy transfer distribution in the *xz*-plane with the ion beam at a lateral width of *x* = 0 nm and the surface position at *z* = 0 nm. (c) Beam diameter *d*_b_(*z*) characterized by the 2σ value of the Gaussian electronic energy loss profile.

Simulations were performed using the monolayer collision mode for 2 × 10^6^ helium ions with a kinetic energy of 15 keV entering the layer system orthogonally to the surface. The resulting penetration depth *z* and the lateral *x*/*z* distributions were extracted from the IONZ3D file of the program (Figure 5b). The lateral energy transfer distribution for each data set on the *z*-axis Θ*_z_*(*x*) was approximated by a Gaussian normal distribution having the form:


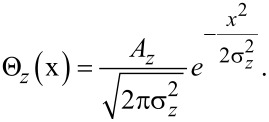


*A_z_* represents the energy loss per depth unit, σ*_z_* is the standard deviation of the normal distribution.

Since the reorientation of the unidirectional EB anisotropy is attributed to local hyperthermal heating by the electronic interaction of the ion beam with the layer system [53], the effective beam diameter *d*_b_(*z*) in the material system is approximated by the 2σ value of the respective normal distribution, *d*_b_(*z*) = 4σ*_z_*.

The simulations show a continuous broadening of the ion beam in the material system and simultaneously a decrease of the transferred energy per depth unit *A_z_*(*z*). The effective beam diameter and the energy loss at the common F/AF-interface where the exchange bias is located are *d*_b_(20 nm) = 27 nm and *A_z_*(20 nm) = 109 eV·nm^−1^·ion^−1^, and at the AF/buffer-interface *d*_b_(50 nm) = 92 nm and *A_z_*(50 nm) = 81 eV·nm^−1^·ion^−1^.

**Figure 6:**
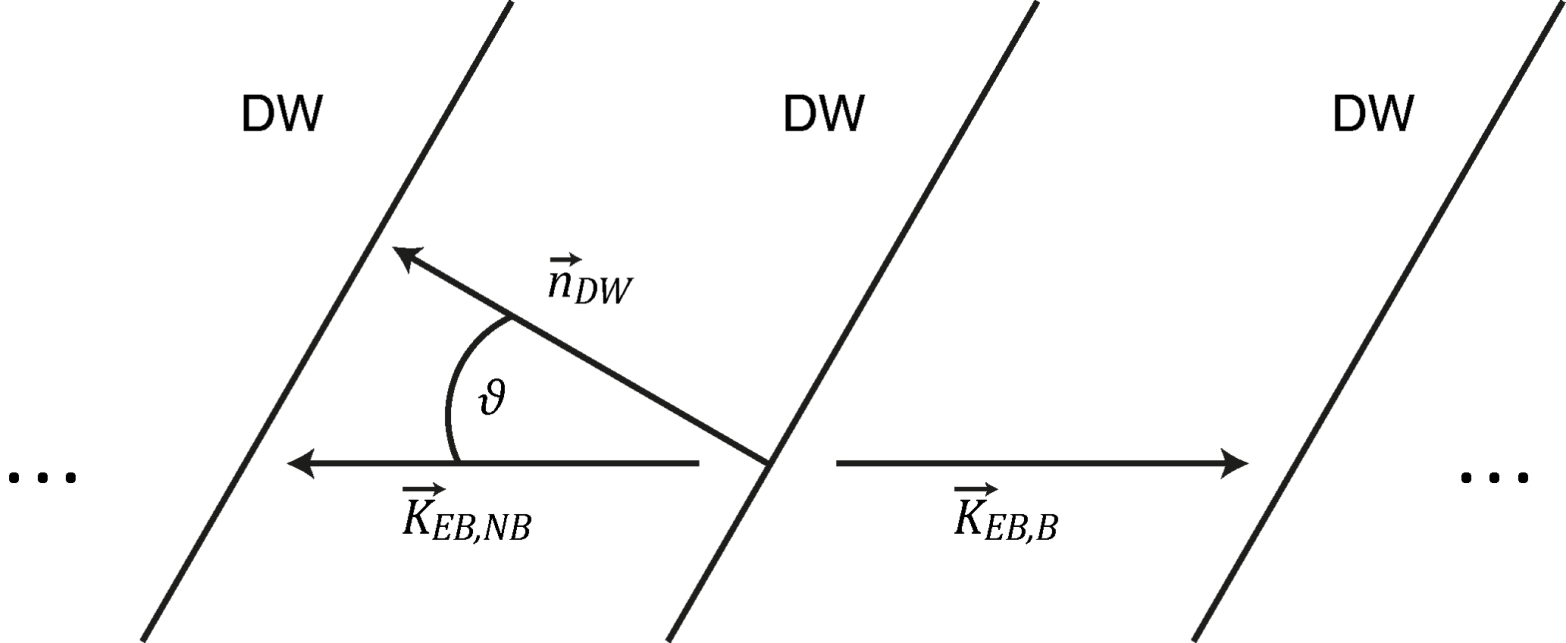
Definition of 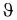. 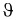 is the angle between the domain wall (DW) normal vector 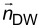 and the local unidirectional EB-anisotropy directions of the non-bombarded (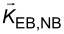) and bombarded (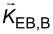) parallel-stripe domains.

**Definition of the angle** 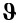
**:** 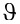 describes the angle between the unidirectional anisotropy axis 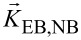 of the non-bombarded domain and the domain wall normal 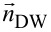 (Figure 6).

**Calculation of the domain wall width:** The formula for the calculation of the domain wall width (Equation 3 in [31]) was modified to:


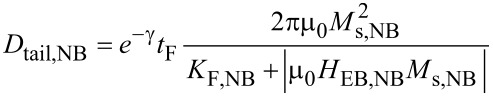


and


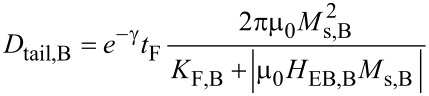


to describe the domain wall tail length of the bombarded and non-bombarded regions individually. Here, γ ≈ 0.577 is the Euler constant, μ_0_ = 4π × 10^−7^ N·A^−2^ is the vacuum permeability, and *t*_f_ is the thickness of the FM layer. The saturation magnetization *M*_s_ as a function of the ion dose was measured in [35] for a similar material system (*M*_s,NB_ = 1226 kA·m^−1^, *M*_s,B_ = 1175 kA·m^−1^ [35]). The F anisotropy constant *K*_F_ a a function of the ion dose was extracted from a fit function in [40]. The relative change of this constant is used in combination with the literature value *K*_F,NB_ = 4.5 × 10^4^ J·m^−3^ [43] and *K*_F,B_ = 0.71*K*_F,NB_ [40]. The exchange-bias fields *H*_EB,NB_ = 12.3 kA·m^−1^ and *H*_EB,B_ = 10.0 kA·m^−1^ were determined from Kerr-microscope measurements. With these values the domain wall tail lengths *D*_tail,NB_ = 1.04 μm and *D*_tail,B_ = 1.32 μm were calculated for the prototypical Co_70_Fe_30_/Ir_17_Mn_83_ layer system. For the Ni_81_Fe_19_ layer system from [29], the values *K*_F,NB_ = *K*_F,B_ = 2.3 × 10^2^ J·m^−3^ [54], *M*_s,NB_ = *M*_s,B_ = 780 kA·m^−1^ [55], *H*_EB,NB_ = −15.5 kA·m^−1^ and *H*_EB,B_ = 13.9 kA·m^−1^ [29] were used to calculate the domain wall tail widths *D*_tail,B_ = 972 nm in the bombarded and *D*_tail,NB_ = 874 nm in the non-bombarded regions.
